# Toward a Local Manipulation of DNA by Quantum Fields

**DOI:** 10.1021/acs.jpclett.6c02166

**Published:** 2026-10-12

**Authors:** Tommaso Cenci, Riccardo Alessandro, Enrico Ronca

**Affiliations:** Dipartimento di Chimica, Biologia e Biotecnologie, Università degli Studi di Perugia, 06123 Perugia, Italy

## Abstract

Hydrogen bonds are the fundamental backbone for deoxyribo-nucleic acid (DNA) stability. In this Letter we investigate a new strategy based on plasmonic cavities to perform manipulation of hydrogen bonds in DNA. The analysis is performed using state-of-the-art Quantum Electrodynamics Coupled Cluster (QED-CC) calculations. We investigate whether in the strong coupling regime modifications of the nucleotide bases’ interactions can be induced in a totally nonintrusive manner. Our strategy proposes an alternative approach to methodologies based on a collective coupling to perform optical DNA manipulation.

## Graphical abstract

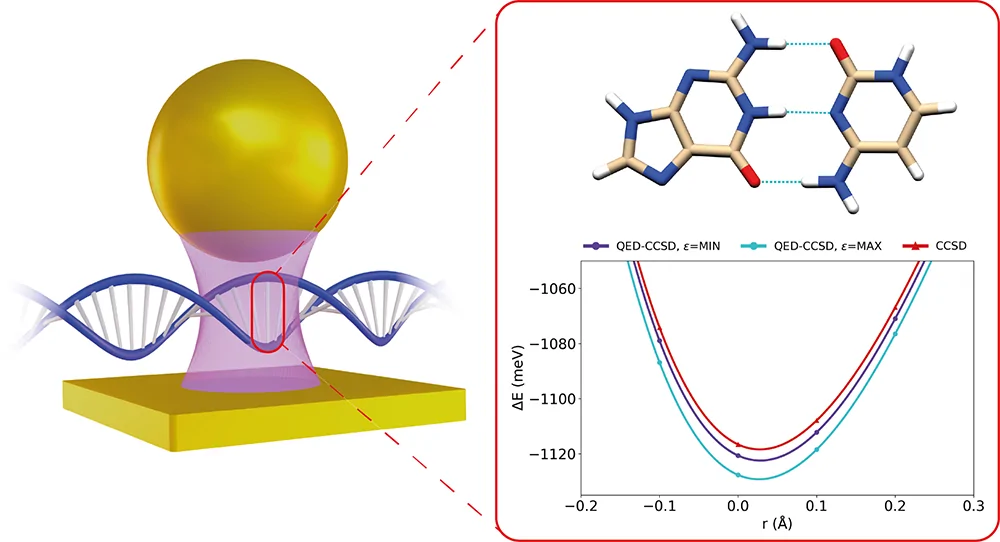

Genetic engineering has been one of the holy grails in biochemistry since its discovery due to its potentially revolutionary applications in many fields ranging from medicine, pharmacy up to industry and agriculture.^1–6^

For these reasons, in the past decade, an exponential increase in both private and public investments dedicated to this sector has been observed and a significant growth has been also foreseen for the near future.^7^ The attention to this field grew also thanks to the advent of accurate experimental techniques to apply local manipulations of deoxyribo-nucleic acid (DNA) strands.^8–11^ Among these techniques, the clustered regularly interspaced short palindromic repeats (CRISPR) approach has revolutionized our capabilities in doing genetic modifications both in vitro and in vivo.^12–16^ The double helix structure of DNA is composed of two antiparallel strands of nucleotides connected by deoxyribose and phosphate groups. Nucleotides pair specifically (adenine with thymine and cytosine with guanine) via hydrogen bonds, representing the main forces that maintain the stability of the whole helicoidal structure.^17–20^ The CRISPR technique uses the insertion of a guide RNA (gRNA) strand in a specific region of the gene. The gRNA activates a nuclease enzyme (i.e., Cas9) able to break double-strands in the selected region. At this moment, the cell activates some repair mechanisms [nonhomologous end joining (NHEJ) or homology-directed recombination (HDR)] responsible for the actual genomic manipulation.^21–27^ Emmanuelle Charpentier and Jennifer Anne Doudna reproduced the CRISPR mechanism in a lab and were awarded the Nobel Prize in Chemistry in 2020.^16^ Despite these advantages, the CRISPR technique suffers from possible off-target or unexpected modifications, limiting its use in several fields of application.^28–30^ For all these reasons, researchers are still dedicating large efforts to formulate novel approaches for DNA manipulation that are able to edit genes in a more precise but, at the same time, less intrusive manner.

In the past decade polaritonic chemistry became very popular in the scientific community as a new tool to manipulate the properties of matter by means of quantum fields confined in optical cavities.^31–34^ Inside an optical cavity (i.e., Fabry–Pérot resonator), the states of the matter couple with the states of the field, generating new hybrid states called polaritons. Polaritons usually have characteristics that can be very different from the bare matter states, offering an unprecedented opportunity to control the properties of matter in a totally nonintrusive manner. The popularity of the polaritonic chemistry field increased enormously thanks to the studies performed by the Ebbesen group, who proposed quantum fields as a new tool to manipulate chemical reactivity. In particular, they demonstrated that Fabry–Pérot cavities set in resonance with specific electronic or vibrational transitions of the reagents can be used to control the chemistry or photochemistry of a reaction.^35–41^ Recently, the Ebbesen group proposed polaritonics as a new technique to destabilize the hydrogen bonds in DNA and induce its melting (opening of the double helix).^42^ Before Ebbesen’s group, the idea of using vibrational strong coupling (VSC) to manipulate biological systems’ properties with a focus on DNA was already proposed by Zhang’s group. In particular, they also observed effects induced by quantum fields on the melting temperature of DNA, attributing the effects to a cooperative VSC with the solvent molecules.^43–47^ In both Ebbesen’s and Zhang’s experiments, strong coupling is reached by collective effects (coupling of many emitters to the cavity field), requiring the use of a macroscopic sample. In these conditions the cavity field has effects on the whole DNA chain, introducing limitations to local control of the modification.

In this Letter, we investigate a new strategy based on plasmonic cavities to manipulate single DNA strands. Inside a plasmonic cavity (see Figure 1) very large coupling values can be reached also for a single (or for a very reduced number) of emitters.^48–50^ In this way, manipulation at the level of the single or few nucleotides pairs is in principle possible. It is important to highlight that differently from the work of Ebbesen and Zhang, our analysis is performed by coupling the cavity field to electronic instead of vibrational states of the DNA strand. The study has been performed by means of theoretical *ab initio* simulations. In order to treat the matter and the field on an equal footing, quantum electrodynamics (QED) extension of quantum chemistry approaches need to be used. In this study, the quantum electrodynamics coupled cluster (QED-CC) theory has been applied.^51,52^ QED-CC is, since its first formulation,^51^ the reference *ab initio* technique for investigating molecular systems strongly coupled to quantum fields. Even though the majority of the established *ab initio* quantum chemistry approaches (i.e., HF,^53–55^ DFT,^56–58^ MP2,^59^ CASSCF,^48,60^ DMRG,^61^ FCI,^51^ etc.) have been extended to QED, coupled cluster is still by far the best compromise between efficiency and accuracy, especially for molecular systems involving weak interactions (i.e., hydrogen bonds, van der Waals, etc.). QED-CC theory has already been used by our group to predict cavity-induced modifications of intermolecular interactions.^62^ In particular, we demonstrated that for this kind of system, an actual polarization-dependent modification of the binding energy can be obtained if sufficiently large coupling values are reached. Theoretically, the potential energy surface reshaping is visible only if correlation effects are included explicitly. This observation justifies the use of QED-CC theory for our investigations of DNA.

**Figure 1:**
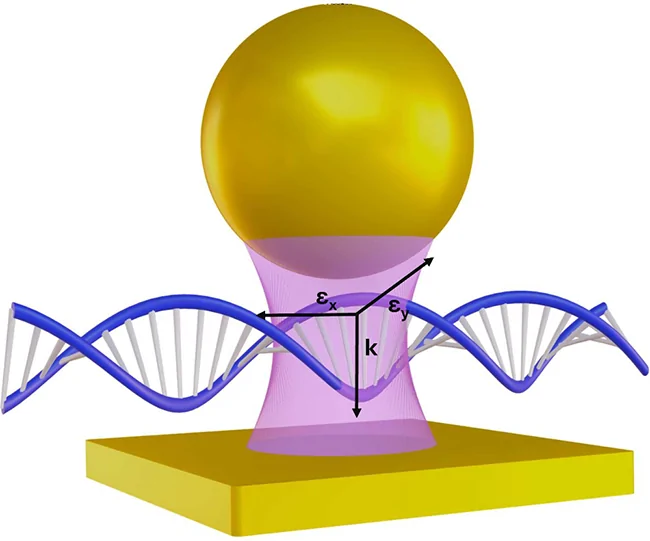
Pictorial representation of a DNA double strand in a plasmonic cavity.

Considering the small dimensions of the plasmonic cavity, our investigations have been performed on single nucleotide bases dimers [adenine-thymine (AT) and cytosine-guanine (CG)] coupled to the electromagnetic field. This approximation can be considered valid since only a single couple (or at most just a few of them) will interact with the field confined inside the device.

The geometries of the dimers (see Supporting Information) have been optimized with the ORCA software package^63,64^ at the MP2 level of theory^65^ using a cc-pVDZ basis set.^66,67^

A coupling value of λ = 0.05 au, achievable only in plasmonic cavities for a single emitter,^48–50^ has been used throughout this Letter. The cavity frequency has been selected to be in resonance with one of the first bright excitations of the nucleotide dimers (5.1 eV for CG and 5.7 eV for AT). These deep ultraviolet plasma resonances values are not accessible in standard plasmonic cavities based on gold or silver (plasmonic excitations up to about 2.4 eV).^68–71^ They might, in principle, be accessed in plasmonic cavities made of different metals (aluminum, rhodium, gallium, etc.),^72–74^ characterized by strong plasma resonances in the 5–6 eV frequency range. However, this would require facing several technical issues (metal oxidation, synthesis of small nanoparticles, etc.).

For all calculations, the single cavity mode approximation has been used.

Inside the plasmonic cavity, the DNA double helix can only lie perpendicular to the wave vector (*k* – set along the *z* axis) of the quantum field as depicted in Figure 1. This implies that only field polarizations (ϵ) on the *xy*-plane are accessible.

All calculations have been performed using the $e^{T}$ software package.^75,76^

Before starting our study, we calculated the Rabi splitting for the AT dimer at a cavity frequency of 6.26 meV setting the polarization direction along (0.97, −0.26, 0). Under these conditions, we obtained an energy separation between the polaritons of about 94 meV. This value, in line with the experimental observations for a single molecule in a plasmonic cavity, validates our choice of the coupling strength.^49^

Now, as a first step, we investigated the effects induced by the field on the hydrogen bonds dissociation potential energy surface as a function of the polarization direction. The analysis has been performed by varying the angle (θ) defining the orientation on the *xy*-plane of the field polarization vector (ϵ) with respect to the *y* axis (see Figure 2).

**Figure 2:**
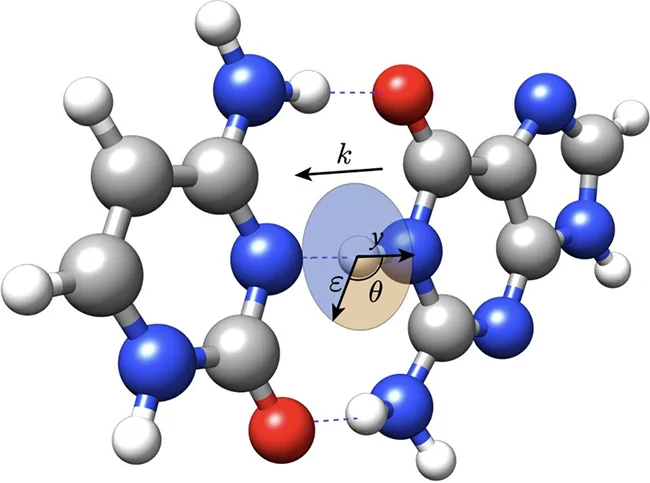
Graphical representation of the angle (θ) defining the polarization direction.

Potential energy surfaces for the hydrogen bonds dissociation outside and inside the cavity with respect to different polarization directions are shown in Figure 3. The selected polarization directions have been chosen for both dimers in situations when the cavity field effect is minimum (MIN) and when it is maximum (MAX) as estimated by the scan reported in Figure S1. Details about the MIN and MAX polarization directions are reported in Table 1 for both nucleotide dimers.

**Figure 3:**
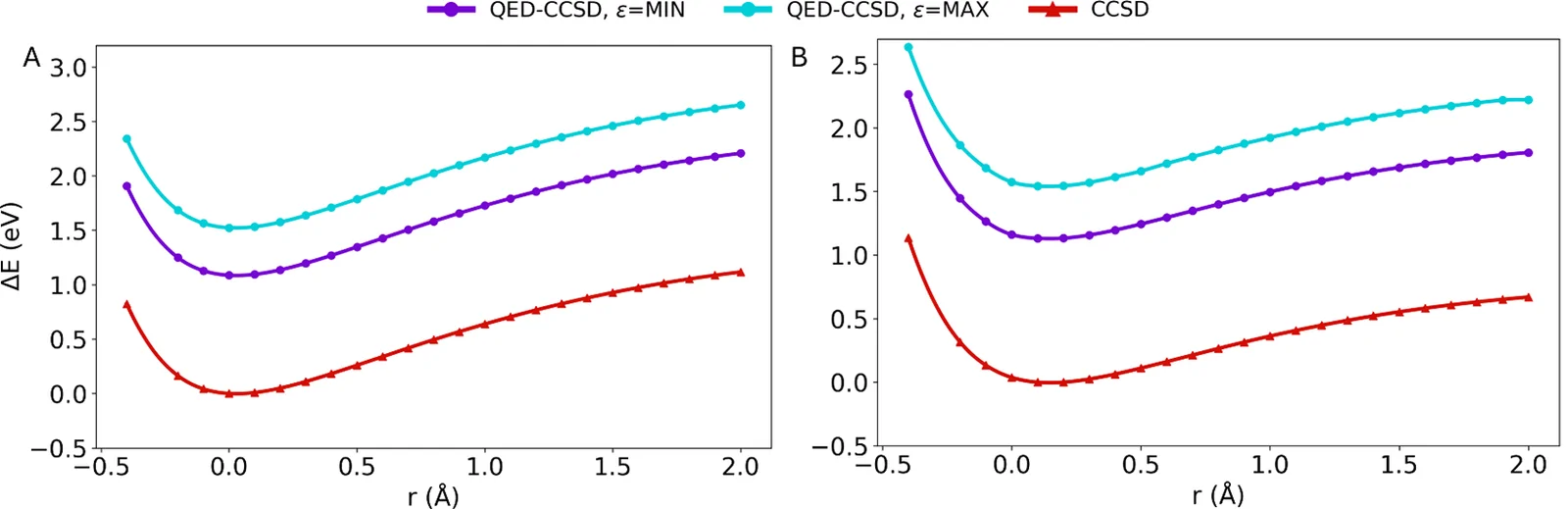
H-bond potential energy curves for the CG (Panel A) and AT (Panel B) dimers outside (red) and inside the cavity. Cyan curves refer to the MAX polarization, while the purple ones refer to the MIN polarization.

**Table 1:** MIN and MAX Polarizations for the CG and AT Dimers

		θ (°)	ϵ coordinates
MIN	CG	0	(0, 1, 0)
	AT	165	(0.26, –0.97, 0)
MAX	CG	90	(1, 0, 0)
	AT	75	(0.97, 0.26, 0)

As a first effect, we notice that, in general, the presence of the field induces an absolute destabilization of the potential energy surfaces. In particular, at the minimum of the potential energy surface, the field-induced energy destabilization is about 1.1 and 1.5 eV for the polarization along the MIN and MAX directions, respectively. Similar effects were observed for both adducts.

A deeper analysis (see Figure 4) highlights that the cavity field, as already observed in ref 62 for a water dimer, can induce also actual modifications of the hydrogen bond energy.

**Figure 4:**
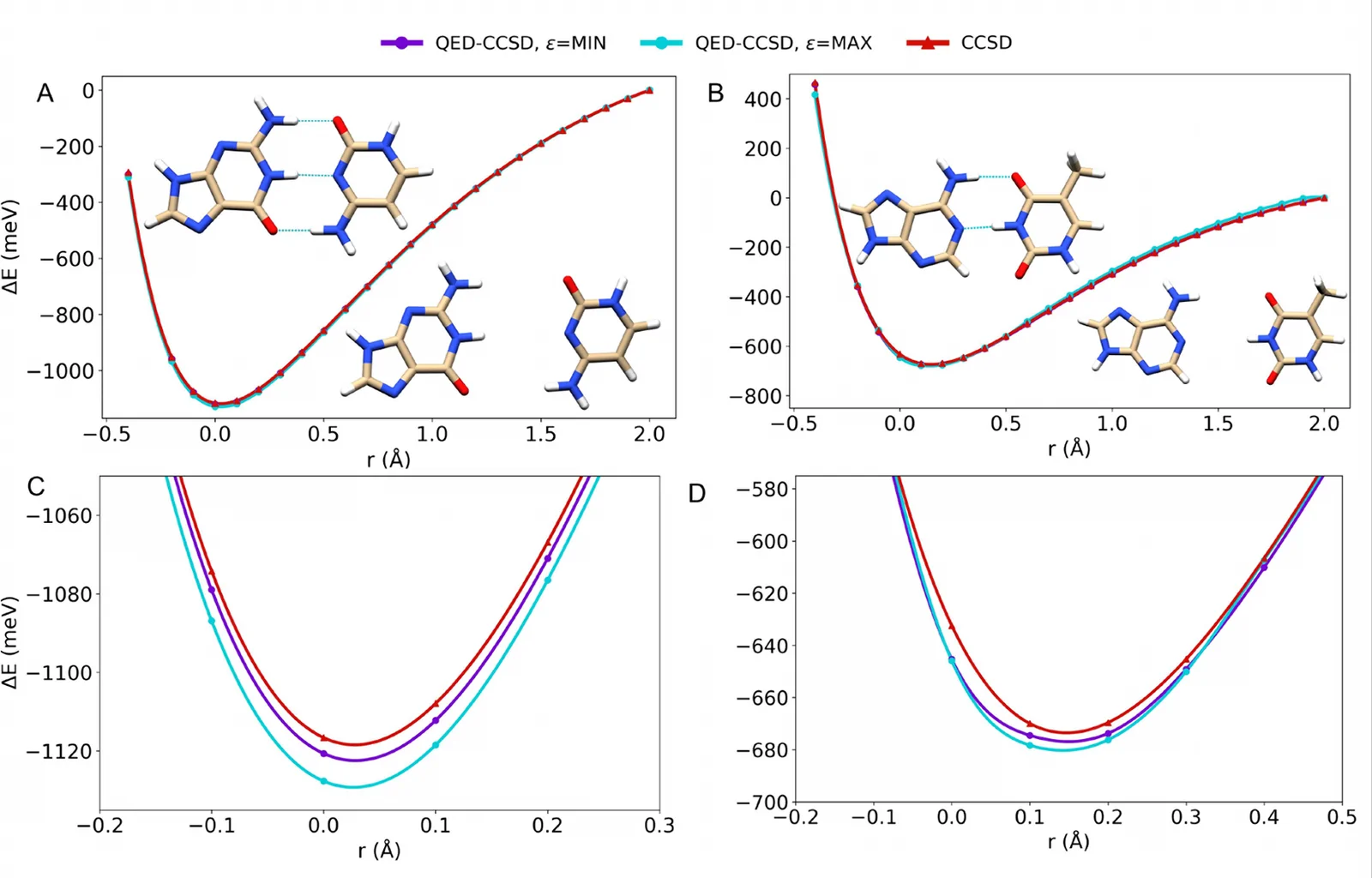
H-bond potential energy curves relative to separated fragments for CG (panel A) and AT (panel B). Panels C (CG) and D (AT) are zooms around the equilibrium distance to better visualize the cavity effects on the two dimers.

This effect can be clearly observed in panels A and B of Figure 4 for the CG and AT dimers, respectively, and highlighted by the corresponding zooms at the energy minima in panels C and D. In particular, differently from the absolute energies, we notice that the field induces a bond stabilization of about 12.4 meV (∼1.1% of the total binding energy) for the CG adduct and 13.5 meV (∼2.1% of the total binding energy) for the AT dimer estimated at the corresponding MAX polarization directions.

The field-induced hydrogen bond stabilization observed in Figure 4 is also different compared to what was observed in ref 62 for the water dimer. Indeed, in that case, the cavity field induced a destabilization of the binding energy. This effect is due to the different alignments between the field polarization and the hydrogen bond. A similar trend was observed in ref 62 for van der Waals interactions in the H_2_ dimer.

To gain deeper insight into this effect, we investigated the field-induced effects on the binding energy as a function of the field polarization direction (or equivalently on the dimer orientation). The results are presented in Figure 5.

**Figure 5:**
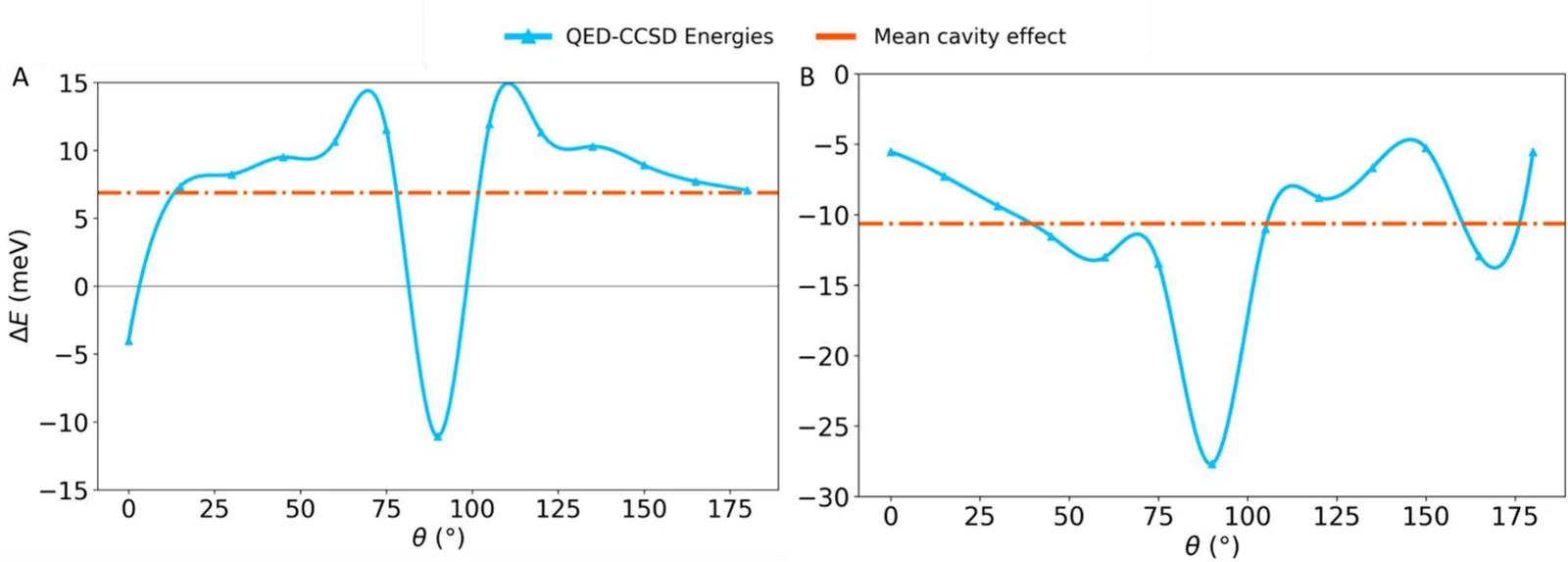
Cavity effects on the binding energies as a function of the field polarization direction θ. Panel A refers to CG, while panel B refers to AT. In panel A, a black horizontal line delimits field-induced stabilizations (positive) from destabilizations (negative).

A different trend can be observed in this case for the two systems. In particular, the CG dimer (see Figure 5A) shows both stabilizing (around 0° and 90°) and destabilizing effects (elsewhere). On the other hand, the AT dimer (see Figure 5B) shows stabilizations for every orientation of the polarization vector. However, it is important to remark that, also in the CG dimer case, the average field effect (orange dashed line) is different from zero. This ensures that the effect does not cancel out in an eventual experiment. For this dimer, the field produces an average destabilizing effect of about 6.7 meV (0.6% of the total binding energy, −1117 meV).

In the AT dimer case instead the average field effect induces a stabilization of the hydrogen bonds resulting in a negative mean cavity effect of about 10.6 meV, 1.68% of the total interaction energy (−632.4 meV). The maximum stabilization, in this case, is about −27.7 meV corresponding to 4.3% of the total binding energy. Because of the significantly lower binding energy, the cavity effect is, in percentage, more pronounced for the AT dimer compared to the CG one.

For this system, we also calculated the hydrogen bond stabilization setting the cavity frequency to 1.9 eV as performed by Chikkaraddy et al. in ref 49. Under these conditions (corresponding to a gold plasmonic cavity), we observe a lower (17 meV) but still appreciable hydrogen bond stabilization. This reduction is probably due to the off-resonance conditions with respect to the AT dimer electronic excitation. In a gold plasmonic cavity, dissipation effects happening on the order of the femtosecond time scale in the deep ultraviolet regime should be significantly reduced. However, it is important to highlight that under all conditions, the effects still remain quite reduced.

Moreover, it is crucial to point out that the coupling value used in this Letter corresponds to a separation between the nanoparticles of about 1.6 nm, which barely fits within the nucleotide dimers. For larger dimensions (1.8 nm) and using the resonant frequency of ref 49, we would obtain a coupling value of 0.005 au, associated with a hydrogen bond stabilization (for the AT dimer) of about 0.13 meV.

The field-induced effects observed on the energies are associated with the corresponding modifications of the electron density of the system. Density differences for the system in the cavity compared to the one outside are presented in Figure S2.

From the figure, we observe that the field tends to localize the charge on the electron-rich regions of the system. The same trend was already observed in previous studies.^51,58,62^

The calculations presented so far have been performed in a dipole approximation and single-cavity-mode framework. To evaluate the effects induced by the inclusion of more cavity modes into the treatment we calculated the potential energy surfaces (see Figure S3) for the AT dimer using a four mode approximation where a wide range of frequencies (0.1, 2.5, 5.71, and 8.9 eV) has been spanned. The polarization of every mode has been selected on purpose on the MAX direction to provide an upper bound to the effect of the additional modes. The obtained results are of the same order of magnitude compared with those evaluated using a single cavity mode. Despite choosing a good number of modes and computational feasibility compromise, our treatment clearly does not include the dense, continuous spectrum of radiative and nonradiative modes present in real metallic nanostructures. The choice of the wide range of frequencies (in particular, the lower ones) has been selected on purpose to include modes belonging also to this continuum. A proper treatment of this aspect would require an explicit (even if approximated) description of the metallic nanoparticles as, for instance, proposed in ref 77. However, this goes beyond the scope of the present Letter. This treatment would also allow us to include, even if in an approximate and purely electrostatic manner, eventual electronic interactions between the molecule and the metallic nanoparticles.

Regarding the dipole approximation, instead, it is only valid if the molecular dimensions are significantly smaller than the size of the device. This is clearly not the case for a plasmonic setup. In order to address the accuracy of our results, we compared the data presented above with those obtained by two modes (+*k* and −*k* with the same resonance frequency) minimal coupling calculations which include the exact exponential spatial dependence of the field. This analysis has been performed using an alternative implementation of the QED-CC approach discussed in ref 78. The resulting tests are presented in Figure S3 of the Supporting Information. For these systems, significant deviations from the discussed trends have not been observed. In particular, minimal coupling effects generate, for the AT dimer, a slightly larger stabilization (compared to the dipole approximation case) of about 20 meV that anyway does not change the general trends observed above.

Moreover, a proper description of DNA nucleotide bases usually requires the use of diffuse basis functions in order to accurately capture the long-range overlap between the electronic densities inside the dimer. This effect might become more evident in the presence of a cavity field. To quantify this aspect we calculated (see Figure S5) potential energy surfaces for the AT dimer using an aug-cc-pVDZ basis set. The obtained results do not show any qualitative change compared to the smaller basis but highlight a relatively large increment of the effects due to both a reduction of the total binding energy (about 30 meV) and an actual increment of the cavity effects (about 1 meV over 13 meV).

In this Letter, we investigated a new approach based on plasmonic cavities to manipulate intermolecular interactions in DNA. The analysis based on QED-CC calculations demonstrated that small modifications of the nucleotide binding energy can be induced by changing the field polarization/DNA strand orientation. In particular, while for the AT dimer a hydrogen bond stabilization is observed for every allowed orientation, for the CG dimer case different orientations of the hydrogen bonds with respect to the field polarization direction can induce stabilization or destabilization of the bond.

This observation brings us to the conclusion that the combination of plasmonic cavities and DNA-origami-based mechanisms^79–82^ able to rotate and immobilize the DNA strand in a specific position might be a good strategy to maximize the cavity-induced effect and to select the orientations at which hydrogen bond destabilization can be obtained.

In the AT case, instead, a reverse approach might be used. In this situation, where only stabilizations are observed, we can imagine using two properly displaced plasmonic cavities (as shown in Figure S4) to stabilize the neighboring nucleotides with consequent destabilization of the selected AT dimer.

Despite its promise, the observed effects are quite small (never exceeding 5% of the total binding energy), with the coupling values (λ = 0.05 au) used in this work. The effects become even smaller if nanoparticle-to-surface distances usually set in the experiments or necessary to host the molecular system are used.

Moreover, it is important to highlight that in our treatment, we partially included dissipation effects only through the choice of the coupling value. A proper treatment of the cavity dissipation effect, very important in highly dispersive devices like those discussed in this work, would require an open quantum system treatment of the effect that is, however, beyond the scope of this Letter. This aspect will be carefully investigated in future work.

Our analysis based on QED-CC calculations highlights new and interesting field-induced effects on the hydrogen-bonding interactions of a DNA strand. However, it is important to clearly stress that the conditions used in our theoretical investigations are, at least for the moment, complicated to reproduce in actual experiments based on plasmonic cavities. This is due to several limitations associated, as discussed above, with the highly dissipative nature of those optical devices, the proper description of the cavity mode continuum, and the need to account for the explicit electronic interaction with the nanoparticles, all aspects that have not been considered in this work.

In conclusion, despite our results being not directly comparable to experimental observations, we think that the proposed strategy might inspire experimentalists toward the fabrication of novel cavity-based devices for DNA manipulation.

## Supplementary Material



## Data Availability

The $e^{T}$ program used to perform QED-CCSD calculations in this work is available at the main software’s GitLab repository: https://gitlab.com/eT-program/eT. The $e^{T}$ code used to perform QED-CCSD calculations with the minimal coupling Hamiltonian can be found at the Zenodo of the original paper (10.5281/zenodo.7035887).^78^ Examples of input files, molecular geometries, and instructions to reproduce the calculations are available at the following Zenodo link: 10.5281/zenodo.11977434.

## Notes

Geometries of the adenine-thymine and cytosine-guanine dimers, plot of absolute energies polarization dependence, plot of cavity induced charge density modifications, and a study of the field spatial dependence effects on the hydrogen bonds dissociation potential energy surfaces (PDF)
