# Supplementary material for: Toward a Local Manipulation of DNA by Quantum Fields
Source: J Phys Chem Lett. 2026 Oct 12;17(42):12111–7. doi: 10.1021/acs.jpclett.6c02166 (PMC13700097; doi:10.1021/acs.jpclett.6c02166)
Supplement: Supplementary file 1 [file jz6c02166_si_001.pdf]

Supporting Information: Toward a local manipulation of DNA by quantum fields

Tommaso Cenci, Riccardo Alessandro, and Enrico Ronca*

Dipartimento di Chimica, Biologia e Biotecnologie, Università degli Studi di Perugia, 06123, Perugia, Italy

E-mail: enrico.ronca@unipg.it

1 Equilibrium geometries

Cytosine-guanine dimer

N	0.0000	-0.0000	1.8717
H	0.0000	0.0000	0.0000
N	-0.0597	0.0757	-1.0368
H	-2.3140	-0.0897	0.9191
O	-2.3511	0.1672	-0.8065
H	2.1774	0.1366	4.3041
N	1.2561	0.0562	3.8829
C	0.1365	-0.0870	4.6473
H	0.2794	-0.1153	5.7310
C	-1.0884	-0.1853	4.0501
H	-1.9979	-0.2982	4.6420
C	-1.1157	-0.1281	2.6038
N	-2.2828	-0.2141	1.9506
H	-3.1451	-0.2589	2.4780
C	1.2264	0.1082	2.4759
O	2.2923	0.2475	1.8670
H	0.3322	0.6021	-5.6932
N	-0.3540	0.5510	-4.9473
C	-1.7274	0.6319	-5.0608
H	-2.2133	0.7712	-6.0269
N	-2.3573	0.5235	-3.8955
C	-1.3259	0.3634	-2.9863
C	-1.3611	0.2016	-1.5545
C	1.1128	0.0913	-1.7600
N	2.2582	-0.1370	-1.0478
H	2.2623	0.0744	-0.0430
H	3.0966	0.1226	-1.5574
N	1.1745	0.2488	-3.0751
C	-0.0689	0.3764	-3.6132

Adenine-thymine dimer

H	0.0000	0.0000	-1.6512
N	0.0000	0.0000	0.0000
H	1.1589	0.2103	4.6081
N	0.3566	-0.0262	3.9770
C	-0.8822	-0.5233	4.2704
H	-1.2088	-0.6916	5.2777
N	-1.6233	-0.7506	3.2266
C	-0.8284	-0.4277	2.1515
C	-1.0312	-0.4187	0.7551
N	-2.1953	-0.6846	0.1559
H	-2.8885	-1.1882	0.6824
H	-2.2237	-0.7222	-0.8662
C	1.1304	0.4070	0.5640
H	1.8989	0.7356	-0.1242
N	1.4127	0.4804	1.8464
C	0.4181	0.0188	2.6137
H	2.0977	0.6327	-5.1599
N	1.1935	0.4475	-4.6564
C	0.0263	0.3167	-5.3607
H	0.1143	0.4787	-6.4252
C	-1.1565	0.0220	-4.7858
C	-2.4248	-0.1202	-5.5638
H	-2.2137	-0.0895	-6.6282
H	-2.9000	-1.0650	-5.3083
H	-3.1144	0.6765	-5.2942
C	-1.1919	-0.1679	-3.3464
O	-2.1840	-0.4870	-2.7022
N	0.0020	0.0731	-2.7052
C	1.2040	0.3653	-3.2763
O	2.2067	0.5565	-2.6103

2 Supporting Figures

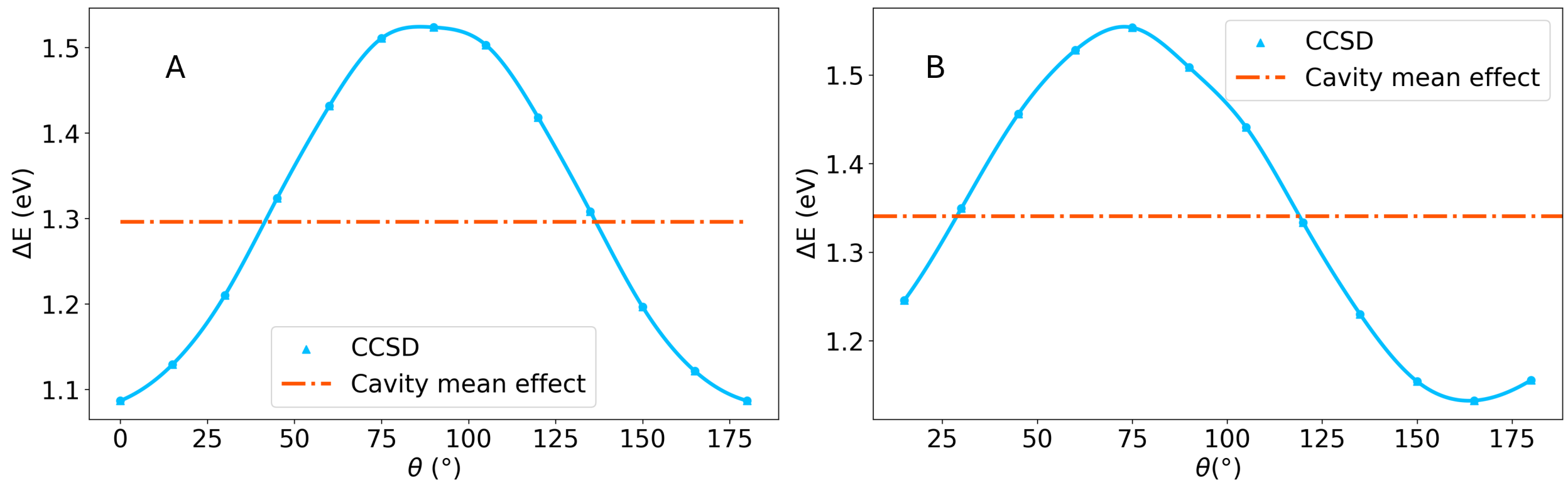

Figure S1: Cavity effect on the CG (panel A) and AT (panel B) absolute energies as a function of the polarization direction.

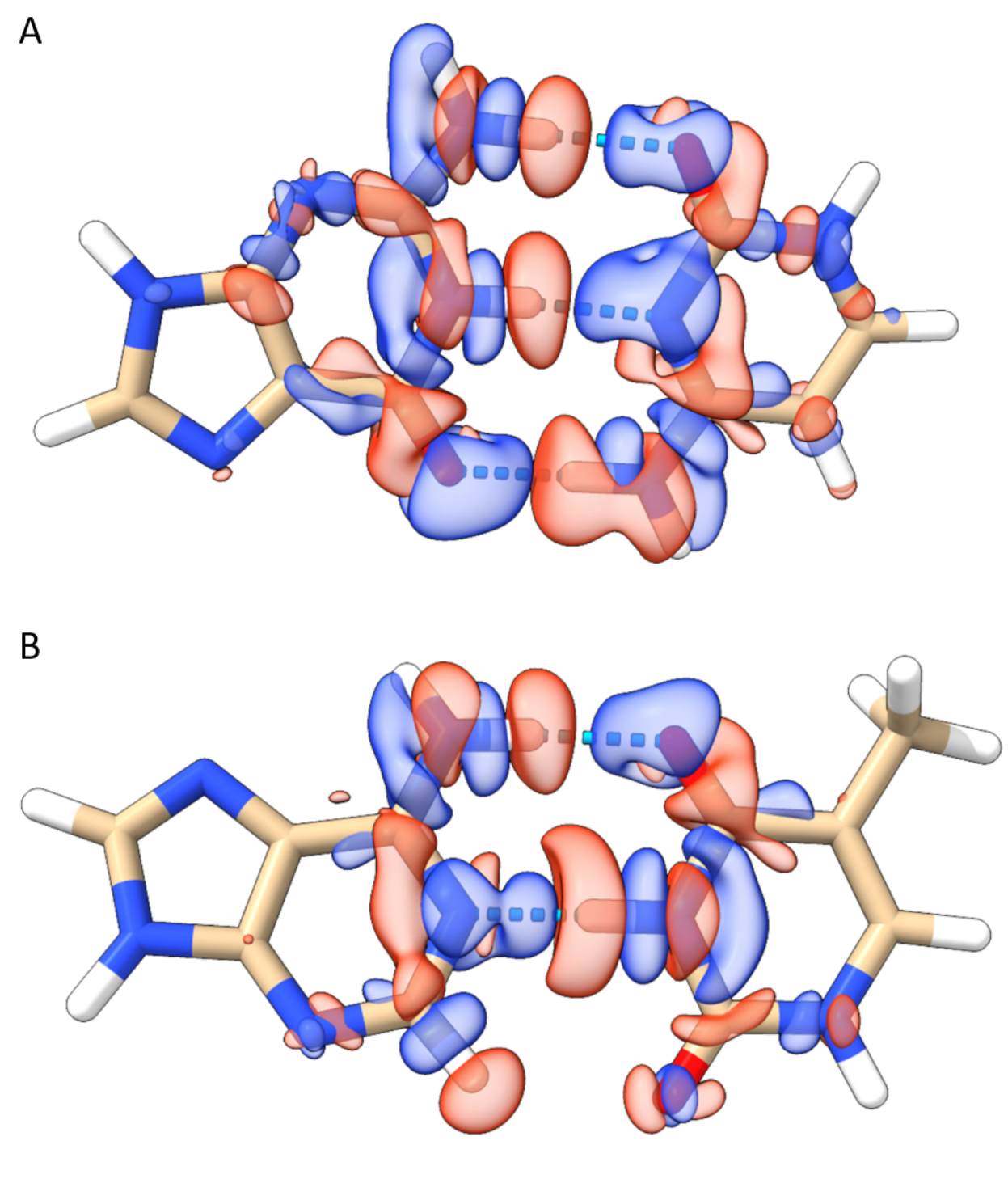

Figure S2: Differences between the electronic densities of the dimers in the cavity ($\theta = 90°$) and the sum of the electronic densities of the separated monomers at the same geometry. Surfaces colored in red represent depletion of charge density, while the blue ones show electron accumulations. Panel A refers to the CG dimer, panel B to the AT one.

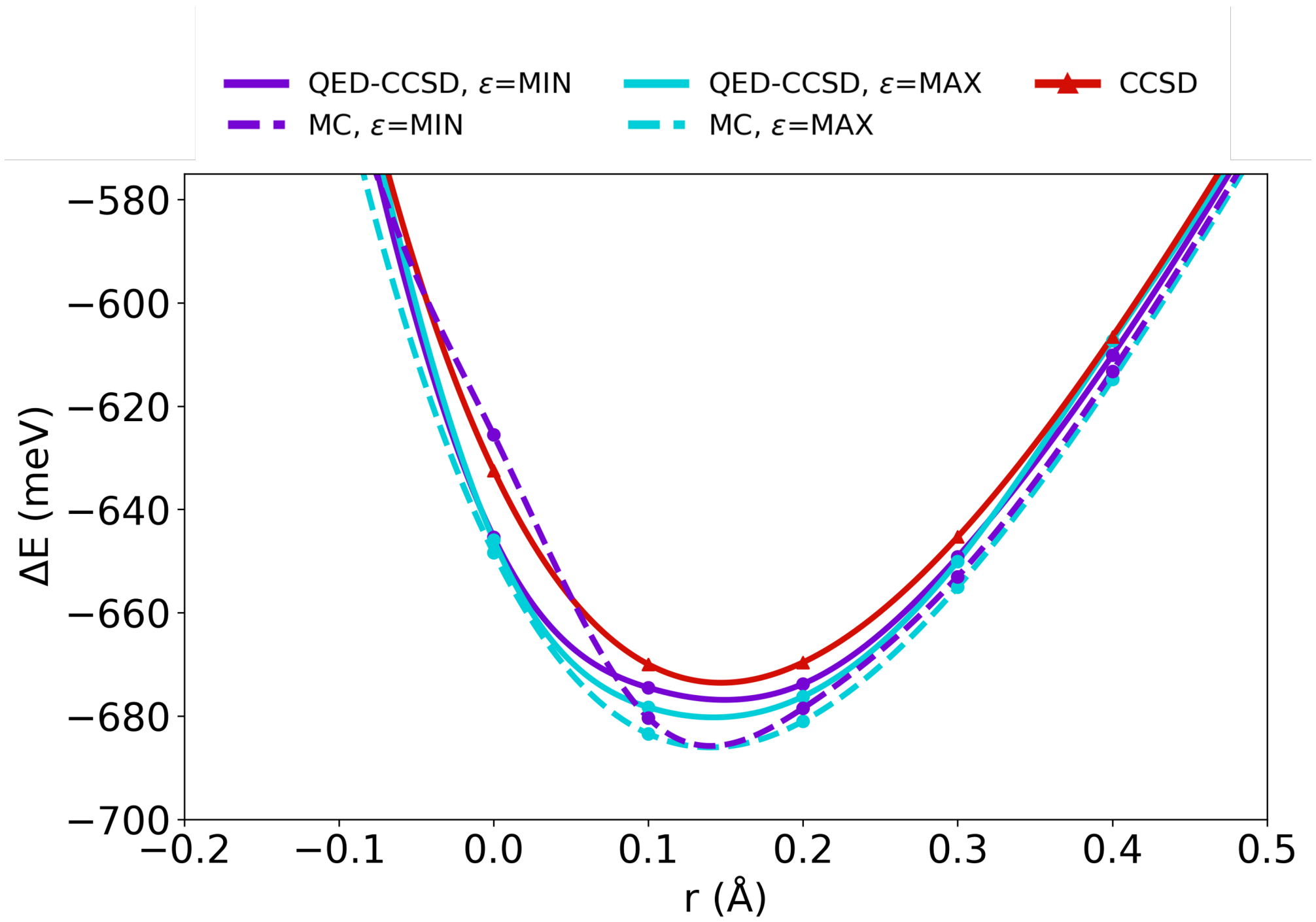

Figure S3: Zoom of H-bond dissociation potential energy curves for AT around the minimum geometry. The curves has been rescaled by the energy of the corresponding dissociated system. Dashed lines refer to QED-CCSD calculations including minimal coupling corrections (depicted as MC).

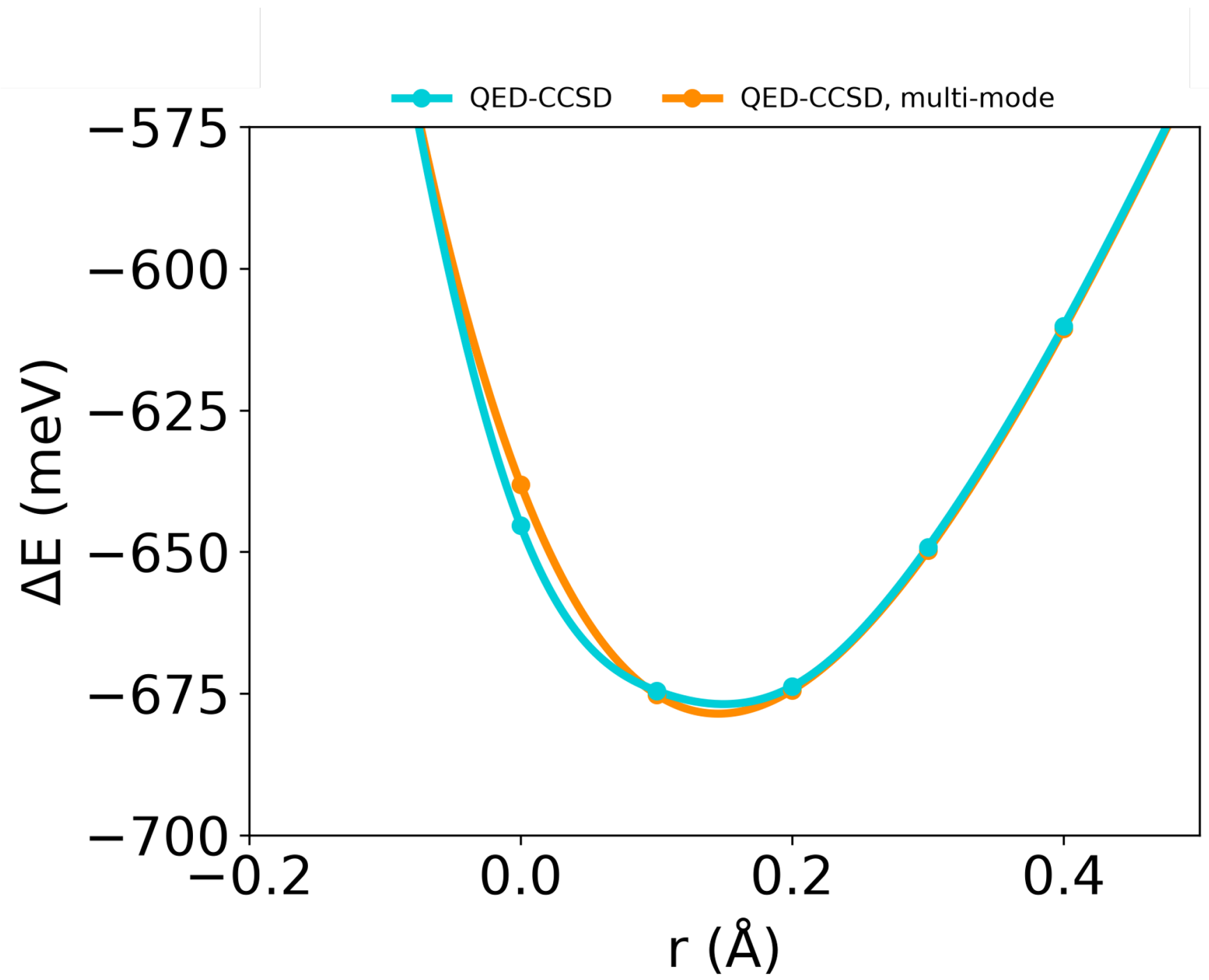

Figure S4: Comparison between QED-CCSD (light blue) and multimode QED-CCSD (in orange) results.

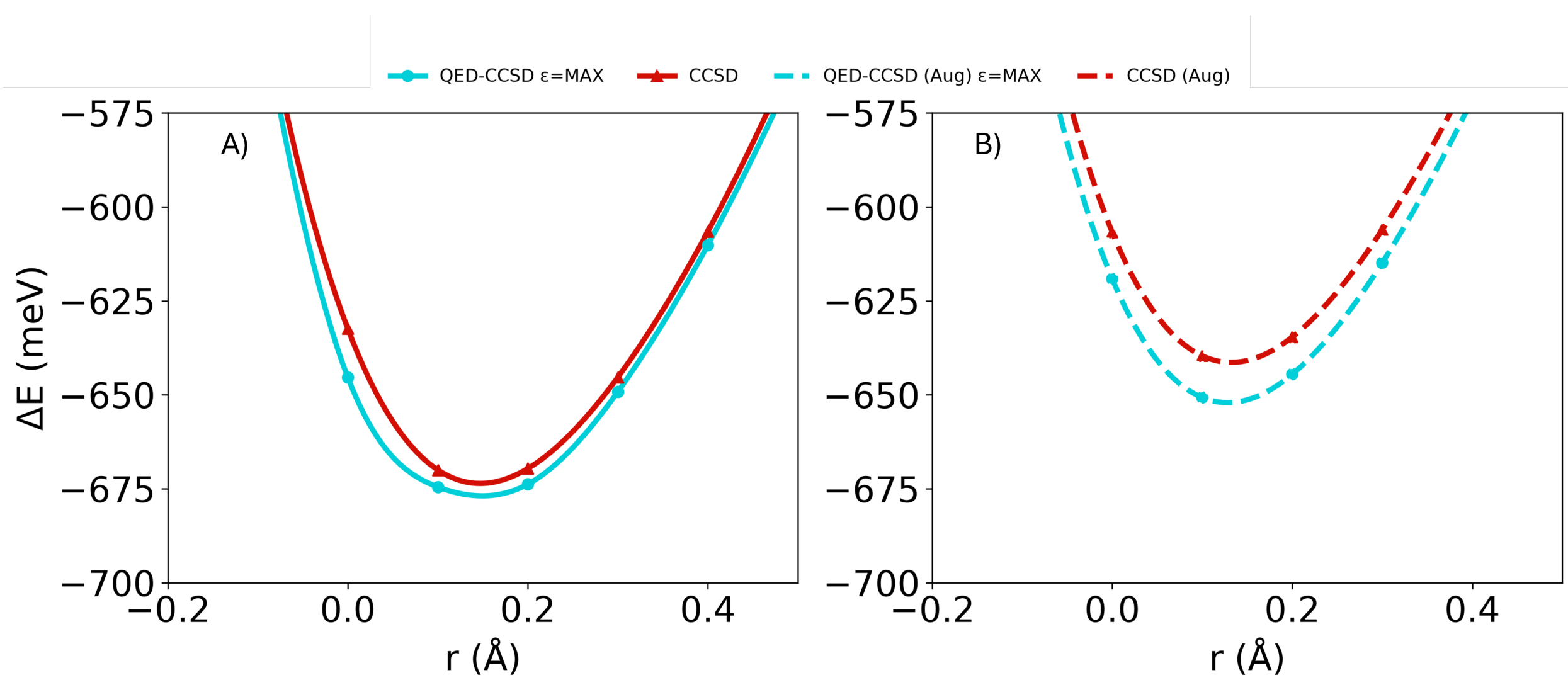

Figure S5: QED-CCSD and CCSD potential energy surfaces around the minimum distance with a cc-pVDZ basis set (Panel A) and aug-cc-pVDZ basis set (Panel B).

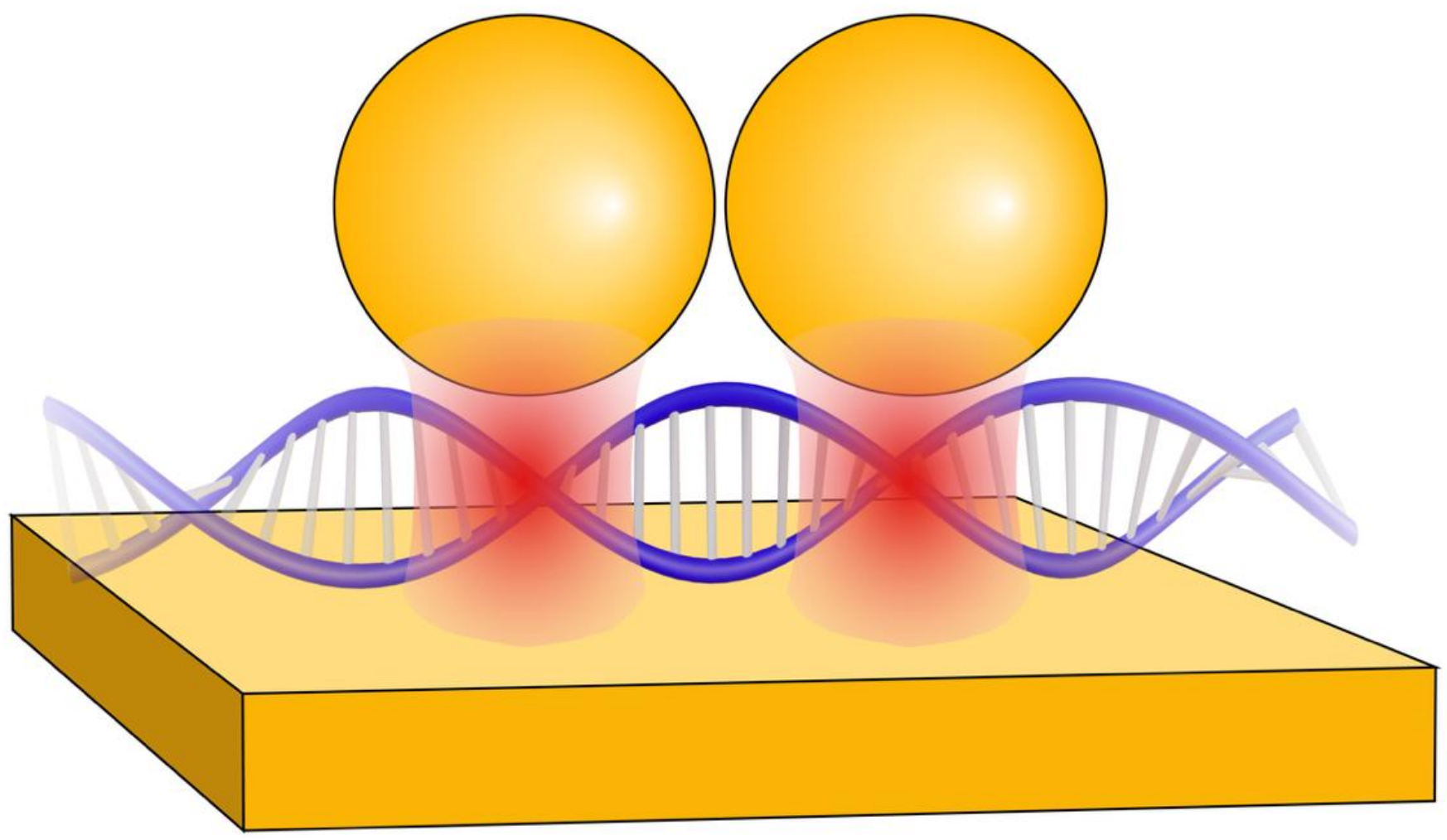

Figure S6: Representation of the double cavity system
